# Preparation and Characterization of Graphene from Refined Benzene Extracted from Low-Rank Coal: Based on the CVD Technology

**DOI:** 10.3390/molecules26071900

**Published:** 2021-03-28

**Authors:** Dun Wu, Meichen Wang, Jiawei Zeng, Jinyuan Yao, Cheng Jia, Hui Zhang, Jiangtao Li

**Affiliations:** 1 School of Earth and Space Sciences, University of Science and Technology of China, Hefei 230026, China; wudun@ustc.edu.cn; 2 Exploration Research Institute, Anhui Provincial Bureau of Coal Geology, Hefei 230088, China; mcwang16@163.com; 3 School of Resources and Environmental Engineering, Hefei University of Technology, Hefei 230009, China; 4 Hefei National Laboratory for Physical Sciences at the Microscale (HFNL), University of Science and Technology of China, Hefei 230026, China; zjw672@mail.ustc.edu.cn (J.Z.); yjyustc@mail.ustc.edu.cn (J.Y.); jiacheng20@mail.ustc.edu.cn (C.J.); 5 CCTEG Shengyang Reserach Institute, Shengyang 110000, China

**Keywords:** refined benzene, distillation, graphene, low-rank coal, CVD, preparation

## Abstract

Industrial preparation of graphene has been a research hotspot in recent years. Finding an economical and practical carbon source and reducing the cost of production and instrument is significant in industrial graphene production. Coal is a common carbon source. Efficient improvement and utilization in the cleaning of coal has recently been a popular research area. In this study, we developed a set of graphene preparation methods based on Anhui Huainan’s low-rank gas coal (HNGC). Using self-built experimental equipment, benzene precursor was prepared from HNGC and used as carbon source to realize graphene growth. The quality of the graphene was characterized by a high-resolution microscope and Raman spectrometer. This study provides a new idea and method for the preparation of low-rank coal-based graphene.

## 1. Introduction

Graphene, as a new type of two-dimensional material, has attracted wide attention from the scientific research community because of its unique crystal structure and electronic properties, which make it have unique electrical, mechanical and thermal properties. Graphene has a good application prospect in various physical and engineering fields. Therefore, obtaining cheap and controllable technology for the industrial preparation of graphene has been widely pursued by the scientific research community. In this area, obtaining a clean and pure carbon source from cheap and common matter to realize graphene is one of the most popular research areas in recent years. Coal is a kind of carbon-containing organic matter with polycyclic aromatic hydrocarbon as the main structural unit, which exists widely in nature. As a big coal country, China is rich in coal resources. Anhui, as a big coal province, has huge coal reserves, and Huainan gas coal is the main coal resource. However, the conversion efficiency of coal is low because the coal is still limited in practical applications of burning. Nowadays, coal, as the main fossil energy in China, will inevitably pollute the environment during processing and utilization. When coal is burned, most sulfur is oxidized to sulfur dioxide (SO_2_), which is discharged with flue gas, polluting the atmosphere and endangering plant growth and human health. Coal mining destroys groundwater resources and exacerbates water shortage in water-deficient areas. Coal mining leads to the emission of waste gas (mainly toxic and harmful gases such as SO_2_, CO_2_ and CO), which harms the atmospheric environment. In addition, there are few inorganic substances in coal, mainly water and minerals, which reduce the quality and utilization value of coal. Therefore, the development of converting coal into clean and efficient materials enhances the utilization value of coal and provides new ideas for tapping the potential industrial value in materials. Among them, the preparation of graphene from coal is a popular research area in this field in recent years [1,2,3,4]. It is of great significance for promoting green energy, creating a series of major innovative products with graphene as the core technology, effectively improving the traditional economic model of coal enterprises and creating green high-tech industries.

As for the research of transforming graphene from coal, it has been reported in China that anthracite and Shanxi lignite are used as carbon sources to prepare graphene. The steps are graphitizing coal first, and then oxidizing and reducing graphite [5]. This method has a low cost in the process of converting coal to graphite, but the graphene produced is of poor quality. The precursor carbon sources of graphene preparation in China are mainly lignite, with low metamorphism and high volatile matter, and anthracite, with high metamorphism and a high degree of internal molecular ordering. For lignite, with low metamorphism, its aromatic lamellar structure is open and disordered with small aromatic lamellar and a large proportion of amorphous structure; for anthracite, it is characterized by a higher directional order of aromatic lamellae and larger aromatic lamellae [6]. Therefore, at present, the technologies are not suitable for Huainan gas coal as the main coal resource in Anhui province. The structural characteristics of Huainan gas coal are a low degree of internal molecular ordering and a loose carbon skeleton. Therefore, it is necessary to treat coal with new technology and produce a new precursor carbon source to prepare graphene. The preparation of graphene from Huainan gas coal can make a new development and breakthrough in the innovative and efficient utilization mode of coal resources in Anhui province.

At present, the method of converting coal into graphene is basically to change large-particle coal into a precursor carbon source before preparing graphene, and the precursor carbon source can be in the form of molecules, or gas. The preparation method of a precursor carbon source includes the preliminary screening of raw coal, impurity removal, pyrolysis (dry distillation), gasification and liquefaction of coal [7,8]. The usual preparation methods of graphene fabrication from precursor carbon sources include chemical synthesis, mechanical exfoliation, pyrolysis of silicon carbide and chemical vapor deposition [9,10,11,12,13,14]. The chemical synthesis method is synthesized by utilizing specific chemical reagents, which is simple to prepare, but the quality of graphene is poor. The mechanical exfoliation method is used to separate graphene layers from highly oriented pyrolytic graphite by special adhesive tape, and finally obtain monolayer graphene because of the weak van der Waals interaction between graphene layers. Graphene obtained by this method has good quality and is suitable to study the physical properties (such as electrical properties) of graphene. However, the amount of graphene generally obtained is small. It is not suitable in the application of industrial production. The pyrolysis method is used to pyrolyze silicon carbide at high temperature, the approach is to sublimate the silicon layer and leave a carbon layer as graphene, but the huge preparation temperature and the high price of silicon carbide single crystal is not suitable for industrial production. The chemical vapor deposition (CVD) method is used to synthesize graphene by chemical reaction and employs a cheap and stabilized catalytic substrate. Following this method, the large area and high-quality graphene can be obtained from the rapid preparation process and inexpensive instrument, which is suitable for industrial production with the prospect in application of converting coal into graphene.

Generally, from the basic principle of chemical vapor deposition (CVD) growth of graphene, polycrystalline copper foil is applied as an economical and efficient catalyst to reduce the decomposition barrier of carbon precursors, and the most common carbon precursor is gaseous methane [10,11,12,13,14]. When the methane molecules are adsorbed on the copper surface, the dehydrogenation is realized by the catalysis of the copper, then the carbon atoms nucleate and finally realize the graphene synthesis.

However, methane is a flammable and explosive gas produced during the coal mining process, which makes the current process complicated and increases the cost of fabrication. In recent years, in order to realize the large-scale industrial production of graphene, more and more new precursor carbon sources have appeared. It is found that graphene can be prepared via some solid and liquid precursor molecules [15,16,17]. This method can greatly reduce the temperature of graphene chemical synthetization and obtain high-quality graphene. When applied to industry, a liquid source is more convenient to store and use than a gaseous source, and the growth mechanism determines that it can grow at a low temperature [18]. Therefore, in terms of energy consumption and convenience, using benzene as carbon source is far more advantageous than using methane as carbon source to prepare graphene, as it can greatly reduce the cost of material preparation. Different from the principle of preparing graphene from methane, when graphene is grown from liquid benzene at high temperature (~1000 °C), benzene molecules are completely decomposed into carbon atoms. The fractured carbon atoms participate in the nucleation of graphene and form uniform monolayer of graphene with high quality. In addition, at a lower growth temperature (300 °C–500 °C), benzene does not need to be completely decomposed, but only undergoes simple dehydrogenation and then bonding. As benzene does not need to break all its chemical bonds in carbon atoms in this process, the absorption energy is low and high-quality graphene is formed, which has considerable potential in the graphene fabrication in a more convenient and economical way [19].

Our study will explore the technology of producing graphene from gas coal to graphene based on the technical route process: from gas coal to coal tar to benzene. That is,

1. Pyrolyzing gas coal in high temperature and oxygen-free environment to produce coal tar;
2. Separating crude benzene and other fractions by distillation;
3. Refining industrial-grade benzene as carbon source to grow graphene.

In view of the mature industrial foundation of the preparation of refined benzene, we focused on the CVD growth of monolayer graphene with benzene as carbon source. For the graphene samples obtained, we used an optical microscope, a scanning electron microscope and Raman spectroscopy to characterize the graphene. In addition, we examined the number of layers and the morphology features of graphene grown under different growth conditions, such as benzene flow rates and growth temperature, in order to explore the possibility of the low-temperature preparation of the graphene in the future.

## 2. Results and Discussion

Coal tar is pyrolyzed from collected gas coal at high temperature under oxygen-free conditions, and then crude benzene is obtained by distillation, and refined benzene with high concentration is obtained by refining. The method of producing graphene with refined benzene as carbon source is as follows: introduce 50 sccm hydrogen gas and keep the tube furnace at 1060 °C for three hours. The main purpose of heating for 3 h with added hydrogen is to remove the oxide on the surface of copper foil, and to make the surface smooth by the reconstruction of atoms within the annealing process. After that, reduce the temperature to 500 °C and reduce the flow rate of hydrogen to 30 sccm (the hydrogen partial pressure, in this case, is 55.6 Pa), meanwhile introduce benzene for 40 min. The flow rate of benzene can be controlled by adjusting the trimming valve in order to avoid the influence and waste caused by volatilization of benzene when the substrate is heated up. Generally, after reaching the growth temperature, the needle valve should be adjusted properly to make benzene fill in the furnace tube at a required rate continuously, which makes the pressure in the tube maintain at the required vacuum condition. After finished the sequence, the ball valve and trimming valve are required to be closed, then the temperature of the tube is cooled down to room temperature naturally. After that, graphene is transferred to silicon oxide by standard wet transfer process.

**Figure 1:**
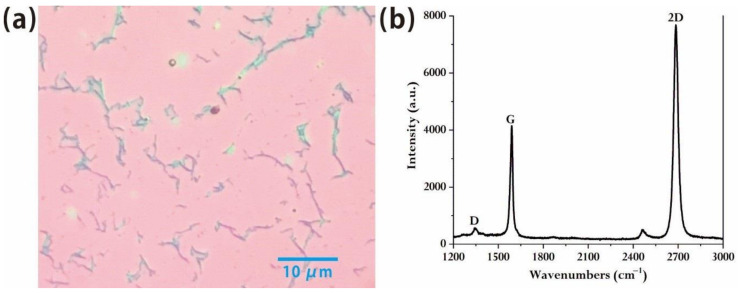
(**a**) Optical microscope diagram of graphene prepared by refined liquid benzene at high temperature of 1060 °C with benzene partial pressure of 1.1 Pa; (**b**) corresponding Raman spectrum diagram of graphene in (**a**).

We found that the growth condition of preparing monolayer and multilayer graphene from benzene at high temperature generally has a large range. Since benzene is liquid, it is hard to control the content of benzene with a traditional flow controller process, so the amount of benzene used is supposed to be accurately adjusted by using a trimming valve. The content of benzene can be controlled and monitored by a vacuum gauge. We control the carbon flux in the growth process by different benzene partial pressures in order to study the graphene quality at different benzene partial pressure. We found that at the partial pressure of benzene of 1.1 Pa, the optical microscopic results show the nearly completely monolayer graphene with a regular and uniform surface. From the image of optical microscopy (Figure 1a), the high quality of samples was shown due to the few textures and large area of the graphene domain. The red part represents monolayer graphene, and the dark part represents the overlap of the graphene which is not completely removed on the opposite side of the copper foil after the transferring process. The density of defects in the material is also very low. The Raman results (Figure 1b) are completely consistent with the Raman peaks of monolayer graphene. They are essentially free of the D peak that is characterized by the quality of graphene following nano-defects [20]. And the intensity of the G peak is close to 1:2 with that of the 2D peak, which also confirms that high-quality monolayer graphene instead of multiple graphene has been obtained [21,22].

**Figure 2:**
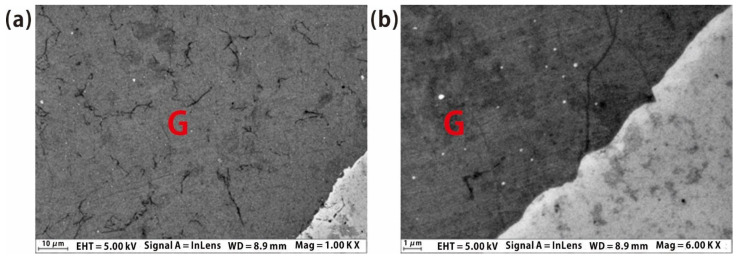
(**a**) SEM image of graphene prepared by liquid benzene at high temperature of 1060 °C with benzene partial pressure of 1.1 Pa. G refers to the parts covered by graphene; (**b**) enlarged SEM image of the graphene surface.

**Figure 3:**
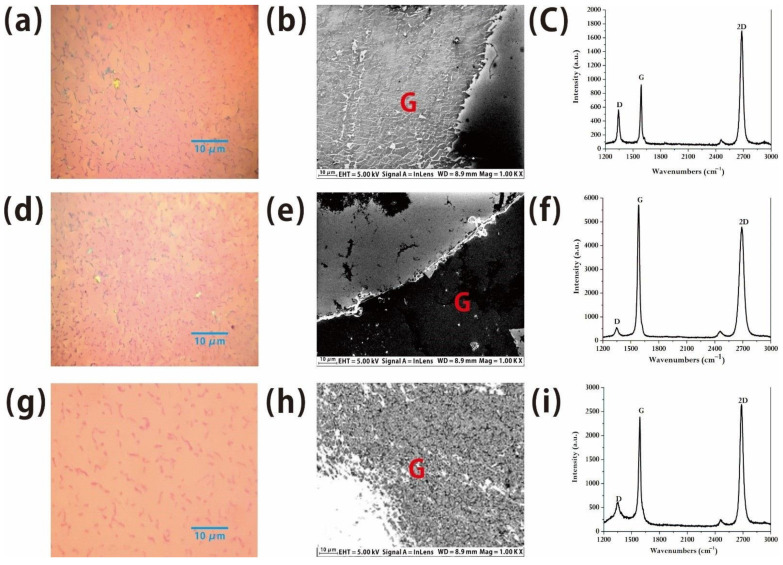
(**a**,**d**) are optical microscopic images of graphene at obtained 1060 °C by liquid benzene (at this time, the pressure of benzene exclusion is 55.6 Pa), and the partial pressures of benzene are 17.3 Pa, 4.4 Pa, respectively. (**b**,**c**,**e**,**f**) are the corresponding electron micrographs and Raman spectra of graphene. (**g**) is the optical microscopic image of graphene obtained at 500 °C by liquid benzene while the partial pressure of benzene is 1.1 Pa, with (**h**,**i**) to be the correspondent electron micrographs and Raman spectra of graphene.

In order to examine the details of the graphene quality, we used the scanning electron microscope (SEM) to show the detailed morphology and the material quality of graphene, as shown in Figure 2a,b. SEM images also show that graphene has extremely high quality, with few domains, defects and impurities. At the same time, the boundaries of graphene are relatively uniform; the degree of the damaged parts and the curl at the graphene edge is extremely low. The zoom-in SEM image also shows the excellent quality (Figure 3b) near the boundary area, without much more domain structure and multilayer graphene on the silicon oxide.

In order to realize the possibility of growing graphene at a low temperature in future, we examined the number of layers and the morphologic features of graphene grown with different growth conditions, such as benzene flow rates and growth temperature. In our experiment, while the partial pressure of hydrogen with a flow rate of 30 sccm was 55.6 Pa, the partial pressure of benzene ranged from 17.3 Pa to 1.1 Pa, and the growth temperature ranged from 1060 °C to 500 °C. When the partial pressure of benzene is 17.3 Pa but the growth temperature is still kept at 1060 °C, it is observed under an optical microscope (Figure 3a) that the prepared graphene has a relatively regular morphology. However, because of the obvious difference in brightness and darkness, it is not difficult to find that the brighter area is mono-layer graphene, while the darker area is the double-layer or multi-layer graphene. We estimated the ratio and found that the ratio of bright area to dark area is nearly 1:1, demonstrating that about half of the areas of the graphene are multiple layer graphene. The detailed image and structure of the results can be observed by SEM (Figure 3b) and there are obvious damaged areas on the surface. At the edge of the graphene, because of this reason, the D peak caused by defects is very high, which indicates that there are a large number of defects in this sample. Due to the large number of defects, the domain wall of the grown graphene/graphite sheet is dense, which makes the graphene seriously damaged at the edge in the transferred process (Figure 3c) with poor thickness uniformity, but we found that the ratio of the intensity of G peak to 2D peak in the Raman spectrum is also nearly 1:2, demonstrating that the monolayer graphene will be obtained at the partial pressure of benzene of 17.3 Pa.

While in order to decrease the amount of benzene, when the partial pressure of benzene is reduced to 4.4 Pa, the quality of the material is obviously decreased and there are some bi-layer/multi-layer graphene structures with the obvious brightness at the edge because of the large thickness from the SEM image (Figure 3e) although the domains in graphite/graphene in the material are greatly reduced. The results of Raman characterization show that the intensity of the D peak is nearly the same and the 2D peak is enhanced with the ratio of I(G):I(2D) = 1.21 (Figure 3f), demonstrating that the proportion of monolayer graphene is lower in comparison with high flow rate benzene [23].

The way to grow graphene at high temperature is to break the benzene ring into carbon atoms and obtain a large area of graphene through the catalytic reaction at copper substrate. However, the benzene ring can directly remove hydrogen atoms and then re-bonds each other to generate monolayer graphene at a low temperature. Following this process, industrial production costs can be greatly reduced. We also prepared graphene at a temperature of 500 °C and the partial pressure of benzene of 1.1 Pa. Under the optical microscope, it was observed that the prepared graphene was very regular (Figure 3g) but there were more textures and domains in the material than the graphene obtained at high temperature. The magnified image of the SEM showed that there were some domain walls and defects on the sample (Figure 3h). Combined with Raman spectroscopy [24], we found that although the morphology characterization has obtained a relatively regular graphene material (Figure 3i), the ratio of G peak to 2D peak is 1.11, indicating that a certain amount of monolayer graphene with multilayer structure are mixed. However, the domain structures are relatively small, and the detailed structure characterization is difficult. Weak D peaks in the Raman spectrum confirmed that there are still some defects in the material. Related experiments also found that when the temperature is between 600 °C and 900 °C, it is difficult to grow into uniform monolayer graphene, for the benzene molecules are not completely decomposed into atoms, but are cracked into some disordered macromolecular chains. At the same time, these macromolecular chains are connected with each other and form graphene films, due to the low migration and diffusion of benzene molecules at a low temperature, thus reducing the quality of monolayer graphene. Since the growth conditions of graphene are closely related to the experimental conditions, high-quality graphene can be obtained by precise control of experiments. How to develop graphene growth at low temperature and achieve good economic benefits will continue.

## 3. Experimental Method

In this study, the coal samples analyzed were from the No. 13-1 coal seam of the Upper Shihezi Formation in Huainan coalfield, northern China. In order to ensure the freshness of the samples, groove sampling was carried out in the underground working face. In the laboratory, vitrified coal strips (removing gangue) were obtained by a manual separation method and they were ground and sieved until the particle size reached 200 mesh. The ground powder sample was then dried and stored in a sealed bag for testing.

The ultimate analysis of Huainan gas coal (HNGC) samples was carried out on a Vario EL element analyzer, German EA Company (Frankfurt, Germany). The contents of carbon (C_daf_, %), hydrogen (H_daf_, %), nitrogen (N_daf_, %), and sulfur (S_daf_, %) were the average of two parallel samples, and the oxygen (O_daf_, %) content was obtained by difference subtraction. According to the GB/T212-2008 standard, the moisture (M_ad_, %), ash (A_ad_, %) and volatile matter (V_daf_, %) of HNGC samples were determined. The experimental results are presented in Table 1.

**Figure 4:**
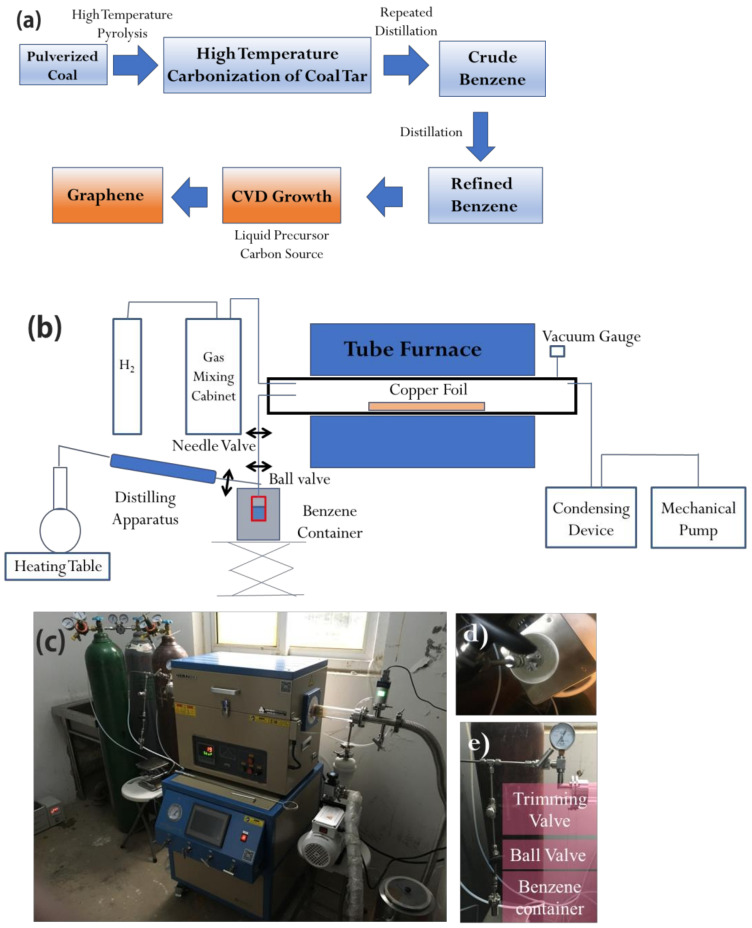
(**a**) Preparation process of graphene with coal as carbon source; (**b**) schematic diagram of the device for preparing graphene from the benzene; (**c**) the photo of the instrument; (**d**) the photo of the benzene container immersed in liquid nitrogen to solidate the benzene inside; (**e**) the photo of the device for controlling the benzene flow rate.

The specific preparation process of this project is shown in Figure 4a, and the process is divided into:

1. Drying distillation of pulverized coal to produce coal tar;
2. Continuously distilling coal tar to obtain crude benzene;
3. Distilling crude benzene continuously to obtain refined benzene;
4. Collecting refined benzene into a liquid pool;
5. Preparing graphene by chemical vapor deposition.

The method of obtaining high-temperature dry distillation coal tar by high-temperature pyrolysis of coal annealing 200 g HNGC to 1000 °C by using a high-temperature pyrolysis program analyzer. The instrument for the preparing of graphene using the CVD method from coal consists of three parts: a tubular furnace of HF-Kejing with a vacuum gauge monitoring the pressure inside, connected to a liquid/gas flow rate control system and a vacuum pump unit (as shown in Figure 4b). The liquid/gas flow rate control system includes a gas circuit connected to the gas mixing cabinet and a benzene container respectively, and both the flow rate of hydrogen and benzene can be controlled in the whole experiment. The gas mixing cabinet is used to control and monitor the flow rate of hydrogen during the CVD process. The liquid flux control system is employed for collecting and controlling the refined benzene (the recovery rate of benzene reaches 99.99%) flow rate. Copper foils are placed in quartz tubes and placed at different positions in a tube furnace to obtain different growth temperatures. Polycrystalline copper with a thickness of 25 μm is generally used as a substrate. For the benzene flow rate in the experiment, the trimming valve and vacuum gauge are used to ensure a proper partial pressure of benzene and a steady flow rate. The final condensing device is used to condense and collect residual liquid benzene (melting point is about 5 °C) with ice for environmental and economic reasons. After adding benzene into the container, it is necessary to solidify benzene with liquid nitrogen and evacuate the air in the container in order to ensure the purity of benzene in the whole experiment [25]. Graphene grown on copper foil needs to be transferred to other substrates. By spin-coating photoresist onto the sample and immersing the sample in 0.1 mol/L ammonium persulfate solution, the copper foil is dissolved, and graphene is transferred to the silicon oxide substrate. Graphene samples are generally characterized by the Raman spectrum with an excitation wavelength of 532 nm, which is a rapid and non-destructive characterization method and very sensitive to the lattice structure of graphene [26]. G peak (1580 cm^−1^), D peak (1350 cm^−1^) and 2D peak (2680 cm^−1^) are caused by defects or disorders that can be used to preliminarily determine the number of graphene layers, the stacking order between layers and the defect and impurity density, respectively. The light transmittance of the optical microscope and the interference of the substrate can be used to directly characterize the graphene layers transferred to the silicon oxide substrate. In addition, the overall roughness and uniformity of graphene can be preliminarily characterized. The scanning electron microscope can be used to characterize the surface morphology details of graphene [27].

## 4. Conclusions

We used coal as a raw material to obtain refined benzene after repeated distillation and refining, and then used it as the precursor of carbon source to obtain monolayer graphene at high temperature (1060 °C) by chemical vapor deposition with different benzene flow rates; the relatively uniform monolayer graphene structure was also obtained by low temperature CVD. Our results show that in future industrial applications, replacing the traditional gas carbon source of methane gas from the Huainan coal with the liquid benzene can produce high-quality monolayer graphene with an economic preparation cost, and has the characteristics of high efficiency and good safety.

Our results also found that the layers and structure of graphene made with CVD process are highly related to the flow rate of benzene. We are working on quantifying the relationship and trying to control the structure and layers. We believe, in future, this method can be used for convenient, flexible and economic growth control of monolayer/multi-layer graphene, which can lay a foundation for the exploration and development of new functions of coal.

## Figures and Tables

**Table 1:** Basic properties of HNGC samples after demineralization.

Proximate Analysis (%)			Ultimate Analysis (%)				
M_ad_	A_ad_	V_daf_	C_daf_	H_daf_	O_daf_	N_daf_	S_daf_
1.37	18.26	33.82	83.3	5.27	9.85	1.23	0.35

ad: air-dried basis; daf: dry ash-free basis.

## Data Availability

The data presented in this study are available in article.

## Sample Availability

Not available.

## Abbreviation

sccm: standard cubic centimeter per minutes.
